# Variation in referrals to secondary obstetrician-led care among primary midwifery care practices in the Netherlands: a nationwide cohort study

**DOI:** 10.1186/s12884-015-0471-x

**Published:** 2015-02-21

**Authors:** Pien M Offerhaus, Caroline Geerts, Ank de Jonge, Chantal WPM Hukkelhoven, Jos WR Twisk, Antoine LM Lagro-Janssen

**Affiliations:** KNOV (Royal Dutch Organisation for Midwives), P.O. Box 2001, 3500GA Utrecht, the Netherlands; Department of Midwifery Science, AVAG and the EMGO Institute for Health and Care Research, VU University Medical Center, P.O. Box 7057, 1007MB Amsterdam, the Netherlands; PRN (the Netherlands Perinatal Registry), P.O. Box 8588, 3503RN Utrecht, the Netherlands; Department of Epidemiology and Biostatistics, VU University Medical Center, P.O. Box 7057, 1007MB Amsterdam, the Netherlands; Radboud University Nijmegen Medical Centre, Internal postal code 118, P.O. Box 9101, 6500HB Nijmegen, the Netherlands

**Keywords:** Midwifery, Referral, Instrumental birth

## Abstract

**Background:**

The primary aim of this study was to describe the variation in intrapartum referral rates in midwifery practices in the Netherlands. Secondly, we wanted to explore the association between the practice referral rate and a woman’s chance of an instrumental birth (caesarean section or vaginal instrumental birth).

**Methods:**

We performed an observational study, using the Dutch national perinatal database. Low risk births in all primary care midwifery practices over the period 2008–2010 were selected. Intrapartum referral rates were calculated. The referral rate among nulliparous women was used to divide the practices in three tertile groups. In a multilevel logistic regression analysis the association between the referral rate and the chance of an instrumental birth was examined.

**Results:**

The intrapartum referral rate varied from 9.7 to 63.7 percent (mean 37.8; SD 7.0), and for nulliparous women from 13.8 to 78.1 percent (mean 56.8; SD 8.4). The variation occurred predominantly in non-urgent referrals in the first stage of labour. In the practices in the lowest tertile group more nulliparous women had a spontaneous vaginal birth compared to the middle and highest tertile group (T1: 77.3%, T2:73.5%, T3: 72.0%). For multiparous women the spontaneous vaginal birth rate was 97%. Compared to the lowest tertile group the odds ratios for nulliparous women for an instrumental birth were 1.22 (CI 1.16-1.31) and 1.33 (CI 1.25-1.41) in the middle and high tertile groups. This association was no longer significant after controlling for obstetric interventions (pain relief or augmentation).

**Conclusions:**

The wide variation between referral rates may not be explained by medical factors or client characteristics alone. A high intrapartum referral rate in a midwifery practice is associated with an increased chance of an instrumental birth for nulliparous women, which is mediated by the increased use of obstetric interventions. Midwives should critically evaluate their referral behaviour. A high referral rate may indicate that more interventions are applied than necessary. This may lead to a lower chance of a spontaneous vaginal birth and a higher risk on a PPH. However, a low referral rate should not be achieved at the cost of perinatal safety.

## Background

In several Western countries, low-risk women can choose to give birth in midwifery settings. If risk factors or complications occur, they will be referred from midwifery care to an obstetric unit. Internationally, most of the referrals during first or second stage of labour (defined as intrapartum referrals in this article) are for non urgent reasons such as a request for pain relief or lack of progress. Nulliparous woman are referred more often than multiparous women [1-8]. This is also the case in the Netherlands [9,10].

Referral rates vary between maternity care settings. Among planned home births the intrapartum referral rates are lower than among planned hospital births or births in midwifery units [11-13]. Additionally, the maternity care system plays a role. A recent review showed that intrapartum referrals rates among planned home births are higher in countries where this service is a regulated part of the maternity care system [14]. In this review, the intrapartum referral rate among planned home births in the Netherlands was the highest of all.

In the Netherlands, independent midwives are the primary caregivers during labour for healthy women with uncomplicated, term pregnancies. These women can opt for a home birth or a hospital birth attended by her own primary care midwife. The attending midwife is responsible for the decision to refer a labouring woman to the obstetric unit, in order to give her access to secondary obstetric care. If birth is planned at home, this referral implies also a transfer to a secondary obstetric care unit. After an intrapartum referral most women will receive fetal monitoring, augmentation of labour, pharmacological pain relief, or a combination of these interventions. Some of them also experience instrumental birth, vaginally or by caesarean section, but most women will still have a spontaneous vaginal birth.

Reasons for referral are listed in the List of Obstetric Indications (*VIL: Verloskundige Indicatie Lijst*), which is regularly updated by a multidisciplinary group of midwives, obstetricians and general practitioners [15,16]. Local protocols developed by midwives and obstetricians are based on the VIL, but may differ in detail. For instance, the VIL recommends a referral to secondary obstetric after 24 hours of ruptured membranes without contractions. It depends on the local collaboration whether this referral takes place in the evening before 24 hours have passed, or the next morning.

Some studies have shown variations in referral rates between midwives in the Netherlands [17,18]. In these studies, midwives’ attitudes towards home birth [18] and the number of midwives in an independent practice [17] have been associated with referral rates.

Although referral in itself is not a negative birth outcome, it is an important intervention in the course of labour and affects the birth experience of women [6,19]. A referral is associated with loss of continuity of care and less sense of control for labouring women [11,20]. Referral might also increase the chance of an instrumental birth for women in primary midwifery care which exposes them to potential side effects.

The background and size of variation in referral rates as well as the consequences for individual women is not fully understood. In this nationwide study, our main goal is to describe the variation in referral rates between all midwifery practices in the Netherlands. Secondly, we want to explore whether a woman’s chance of an instrumental birth is affected by the referral rate of her midwifery practice.

## Methods

### Population and measures

In the Netherlands, births are registered in four databases: one for primary care midwifery (national perinatal database-1), one for the small group of general practioners who provide primary maternity care (national perinatal database-h), one for secondary obstetric care (national perinatal database-2), and one for pediatric care (national neonatal register). These databases are combined using a validated linkage method into the national perinatal database [21,22]. The resulting database contains > 96% of all births in the Netherlands [23]. For this study we used data from all primary care midwifery practices that contributed to the national perinatal database in each year in the period 2008–2010. We included births of women who were in primary care at the onset of labour and who gave birth at term (gestational age 37 weeks or more). Women with a known risk of complications, such as multiple pregnancy or a previous caesarean section, are referred antenatally and are in obstetrician-led care at the onset of labour. As a result, births in our study can be considered as low risk at the onset of labour.

The primary outcomes were referral rates in midwifery practices, and instrumental birth (forceps or vacuum birth or unplanned caesarean section). Secondary outcomes were obstetric interventions during labour (augmentation with oxytocin and pharmacological pain relief), postpartum hemorrhage (PPH) > 1000 ml, and an Apgar score at 5 minutes <7 and <4.

The intrapartum referral rate for each practice was calculated as the number of referrals during the first or second stage of labour divided by the total number of births attended by the midwifery practice. Since parity is strongly related to the chance of an intrapartum referral, and the proportion of nulliparous women is likely to be different per midwifery practice, we also calculated the intrapartum referral rates for nulliparous and for multiparous women separately. Referrals were classified into urgency categories (urgent, non-urgent first stage or non-urgent second stage). A referral was classified as urgent if the referral reason was for a complication that requires immediate investigation or treatment in secondary obstetric care, such as suspected intrapartum fetal distress or placental abruption. A referral was considered to be non-urgent if the referral was for a situation that requires diagnostics or treatment in secondary obstetric care, but without emergency. Examples of non-urgent referral reasons are a request for pharmaceutical pain relief, meconium stained liquor without other signs of fetal distress, and lack of progress [9,10].

The median number of births attended in the three years of the study period was used as a measure for the size of each practice. Information on the number of midwives in the practice is not available in the national perinatal database.

The following maternal characteristics that were registered in the national perinatal database and that might be associated with the chance of referral or of an instrumental birth were identified: parity (nulliparous versus multiparous), maternal age (<25; 25–34; ≥35 year), background (Dutch; non Dutch) and planned place of birth (home; hospital; other/unknown). Social economic status (SES) and level of urbanization were recorded, based on the four digits of the postal code.

The presented data are anonymised and cannot be related to individual women or midwifery practices. The privacy committee of the Netherlands Perinatal Registry approved this study. Further consent and ethical approval is not needed in the Netherlands for this type of study.

### Analysis

We calculated means and standard deviations for intrapartum referral rates in the practices, overall and for nulliparous and multiparous woman separately. To verify whether low (or high) referral rates in a practice affected both nulliparous and multiparous women, the correlation between nulliparous and multiparous intrapartum referral rates per practice Pearsons’ Rho was computed. Practices’ referral rate for nulliparous women was highly correlated with their referral rate for multiparous women (Pearson’s rho .650, p < .001), as well as with their overall referral rate (Pearson’s rho .863, p < .001). We used the nulliparous intrapartum referral rate in the further analyses, and divided the practices into three tertile groups with a lower, intermediate and higher rate of nulliparous referrals.

The association between the level of referrals in a practice and a woman’s individual chance of an instrumental birth was examined using multilevel multivariable logistic regression to take into account clustering of maternal characteristics in midwifery practices. Models were built for nulliparous and multiparous women separately. The independent variable in each model was the level of nulliparous intrapartum referrals in the midwifery practice. The tertile group T1, with the lowest rate of referrals, was the reference category. The dependent variable was instrumental birth (yes/ no).

In the multilevel multivariable logistic regression procedure, models were first adjusted for confounding by maternal characteristics (maternal age, gestational age, ethnic background, urbanisation, SES). After that, we entered planned place of birth, practice size, and receiving a labour intervention one by one, to assess the impact of each factor on the individual chance of an instrumental birth. Results were expressed as odds ratios (OR) and 95% confidence intervals.

Descriptive statistics and bivariate analyses were performed in SPSS 20.0 (SPSS, Chicago, IL USA). The multilevel analyses were performed in Stata version 9.0 (Tata Corp., College Station, Texas, USA). A p-value of < 0.05 was considered as statistically significant.

## Results

### Variation in referral rates

**Figure 1:**
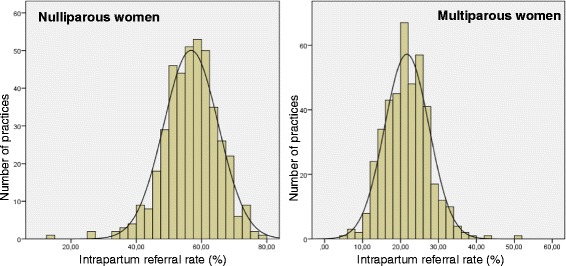
**Variation in intrapartum referrals in 421 midwifery practices in the Netherlands.** Left Nulliparous women; right: Multiparous women.

The cohort included 421 primary care midwifery practices and a total number of 242,965 births. The overall intrapartum referral rate varied between practices from 9.7 to 63.7 percent (mean 37.8; SD 7.0). For nulliparous and multiparous women practices’ referral rates varied from 13.8 to 78.1 percent (mean 56.8; SD 8.4), and from 5.3 to 50.7 percent (mean 21.7; SD 5.9) respectively (Figure 1).

Practice size and distribution of parity in the three tertile groups of nulliparous referral rates are displayed in Table 1. The distribution of maternal characteristics is presented in Table 2.

**Table 1:** **Practice characteristics**

		**Rate of intrapartum referrals among nulliparous women in midwifery practices (tertiles)**				
		**T1 (low)**	**T2**	**T3 (high)**	**Total**	**χ** ^**2**^ **p-value**
		**n = 140**	**n = 141**	**n = 140**	**n = 421**	
Practice size	≤ 139	57	38	46	141	.19
(nr of births attended/yr)	140-229	42	50	47	139	
	≥ 230	41	53	47	141	
Distribution of parity	Nulliparous women	46.0%	46.4%	46.8%	46.4%	
	Multiparous women	54.0%	53.6%	53.2%	53.6%

**Table 2:** **Maternal characteristics by intrapartum referral rates in practices**

			**Rate of intrapartum referrals among nulliparous women in midwifery practices (tertiles)**			
		**T1 (low)**	**T2**	**T3 (high)**	**Total**	
**Nulliparous women**		**n = 35,180**	**n = 40,021**	**n = 37,605**	**n = 112,806**	**χ** ^**2**^ **p-value**
Maternal age	<25	21.1	19.3	18.9	19.7	**<0.001**
	25-34	69.2	70.7	70.9	70.3	
	≥35	9.7	10.0	10.3	10.0	
Gestational age	37-41 + 0	82.8	82.9	83.2	82.9	**0.025**
	>41 + 0	17.2	17.1	16.8	17.1	
Background	Dutch	80.8	84.9	82.3	82.8	**<0.001**
	Not Dutch	19.2	15.1	17.7	17.2	
SES	Cat 1 (high)	21.2	23.2	23.0	22.5	**<0.001**
(Social economic status)	Cat 2	45.7	44.3	43.6	44.5	
	Cat 3 (low)	33.1	32.5	33.4	33.0	
Urbanization	Very urban	25.0	21.9	19.6	22.1	**<0.001**
	Intermediate	51.4	60.1	63.4	58.5	
	Rural	23.6	18.0	16.9	19.4	
Planned place of birth	Home	44.4	42.6	39.9	42.2	**<0.001**
	Hospital	44.9	48.8	51.6	48.5	
	Other/unknown	10.6	8.7	8.6	9.2	
**Multiparous women**		n = 41,273	n = 46,158	n = 42,708	n = 130,139	
		%	%	%	%	
Maternal age	<25	6.2	5.8	5.6	5.9	**<0.001**
	25-34	68.7	68.2	68.8	68.6	
	≥35	25.1	26.0	25.5	25.6	
Gestational age	37-41 + 0	83.9	83.5	84.2	83.9	**0.001**
	>41 + 0	16.1	16.5	15.8	16.1	
Background	Dutch	80.3	82.6	78.9	80.6	**<0.001**
	Not Dutch	19.7	17.4	21.1	19.4	
SES	Cat 1 (high)	22.6	25.4	25.1	24.4	**<0.001**
	Cat 2	47.9	45.7	44.6	46.0	
	Cat 3 (low)	29.4	28.9	30.4	29.5	
Urbanization	Very urban	19.3	16.3	14.9	16.8	**<0.001**
	Intermediate	52.9	62.6	65.7	60.5	
	Rural	27.9	21.1	19.3	22.7	
Planned place of birth	Home	52.0	50.1	46.9	49.6	**<0.001**
	Hospital	37.4	41.3	44.4	41.1	
	Other/unknown	10.6	8.6	8.8	9.3	

Missing values: parity 20, maternal age: 213; background: 2,350; SES 5,896, urbanization 286.

Although the differences between tertile groups are small, the practices in the lowest tertile group (T1) had a somewhat more favourable composition of their client population in some aspects. For instance, in these practices more women planned a home birth in comparison with the total population (nulliparous women: 44.4% versus 42.2%; multiparous women 52.0% vs 49.6%), and more women lived in a rural area (nulliparous women: 23.6% versus 19.4%; multiparous women 27.9% versus 22.7%). In other aspects the client population in these practices was less favourable compared to the total study population. More women had a background that was not Dutch (nulliparous women 19.2% versus 17.2%; multiparous women 19.7% versus 19.4%), and more women lived in a very urban area (nulliparous women: 25.0% versus 22.1%; multiparous women 19.3% vs 16.8%).

### Referrals, interventions and birth outcomes

Table 3 shows referrals, interventions and birth outcomes in the three tertile groups of practices. The largest difference in referrals between practices in the lowest tertile group (T1) versus the highest tertile (T3) group was for non-urgent reasons in the first stage (33.6% versus 48.0% for nulliparous women, and 13.5% versus 20.5% for multiparous women). Differences in urgent referrals were found as well (2.7% in T1 versus 3.8% in T3 for nulliparous women, and 0.9% in T1 versus 1.4% in T3 for multiparous women).

**Table 3:** **Referrals, interventions and birth outcomes in women in primary care, by intrapartum referral rates in practices**

	**Rate of intrapartum referrals among nulliparous women in midwifery practices (tertiles)**				
	**T1 (low)**	**T2**	**T3 (high)**	**total**	**χ** ^**2**^ **p-value**
**Nulliparous women**	**n = 35,180**	**n = 40,021**	**n = 37,605**	**n = 112,806**	
**Referral type**	%	%	%	%	
No dp referral	51.9	42.8	35.2	43.1	
Non urgent 1st stage	33.6	42.1	48.0	41.4	
Non urgent 2nd stage	7.5	8.9	9.5	8.7	
Non urgent, stage unclear	4.3	3.1	3.5	3.6	
Urgent	2.7	3.1	3.8	3.2	
**Obstetric intervention during labour** ^#^					
None	65.4	58.4	51.7	58.4	**<.001**
Pain relief (no epidural)	12.6	15.7	15.8	14.8	
Epidural (1st stage)	11.7	14.0	19.3	15.1	
Augmentation	28.6	34.2	39.8	34.3	
**Mode of birth**					**<.001**
Spontaneous vaginal	77.3	73.5	72.0	74.2	
Instrumental vaginal	15.3	18.0	18.8	17.4	
Caesarean section	7.3	8.6	9.2	8.4	
**Morbidity**					
PPH > 1000 cc	5.3	5.6	6.2	5.7	**<.001**
Apgar score (5 min) < 7	1.2	1.0	0.9	1.0	**.019**
Apgar score (5 min) < 4	0.3	0.2	0.2	0.2	**.030**
**Multiparous women**	n = 41,273	n = 46,158	n = 42,708	n = 130,139	
**Referral type**	%	%	%	%	
No dp referral	82.2	78.8	74.3	78.4	
Non urgent 1st stage	13.5	16.6	20.5	16.9	
Non urgent 2nd stage	1.3	1.7	1.7	1.6	
Non urgent, stage unclear	2.1	1.7	2.1	2.0	
Urgent	0.9	1.2	1.4	1.1	
**Obstetric intervention during labour** ^#^					
None	90.7	89.3	86.4	88.8	**<.001**
Pain relief (no epidural)	3.6	4.4	5.4	4.5	
Epidural (1st stage)	1.4	1.5	2.3	1.7	
Augmentation	7.3	8.2	10.2	8.6	
**Mode of birth**					.234
Spontaneous vaginal	97.3	97.2	97.2	97.2	
Instrumental vaginal	1.5	1.7	1.6	1.6	
Caesarean section	1.2	1.1	1.2	1.2	
**Morbidity**					
PPH > 1000 cc	3.3	3.5	3.9	3.6	**<.001**
Apgar score (5 min) < 7	0.5	0.4	0.4	0.4	.390
Apgar score (5 min) < 4	0.1	0.1	0.1	0.1	.311

Missing values: parity 20, referral type 1; obst. interventions 5; Mode of birth 1,294; PPH 2,374; Apgar score 98.

^#^sums up to >100%, more than one intervention possible.

Both pain relief and augmentation were less often used in the lowest tertile group. More nulliparous women had a spontaneous vaginal birth compared to the middle and highest tertile group (T1: 77.3%, T2:73.5%, T3: 72.0%). Both an instrumental vaginal birth (15.3% versus 18.0% and 18.8%) and a caesarean section (T1: 7.3%, T2: 8.6%, T3: 9.2%) were less often performed in this group. These differences in mode of birth were statistically significant (Chi Square p-value < 0.001). For multiparous women there were no significant differences in mode of birth. More than 97 percent experienced a spontaneous vaginal birth in all tertile groups.

Nulliparous and multiparous women had a PPH > 1000 ml less often in the lowest tertile group. Among nulliparous women, a low Apgar score happened more often in the lowest tertile group, although the prevalence was low in all groups (AS <7: T1 1.2%, T2 1.0%, T3 0.9%; AS < 4: T1: 0.3%, T2 and T3: 0.2%).

In Table 4 the associations between the referral rate in the practice and the chance of an instrumental birth are presented. Nulliparous women in practices in the middle or highest tertile group had a higher chance of an instrumental birth compared to women in practices in the lowest tertile group. (T2: OR 1.22; CI 1.16-1.31; T3: OR 1.33; CI 1.25-1.41). For multiparous women, no significant association was found.

**Table 4:** **Multilevel logistic regression: rate of referrals in a practice and the chance of instrumental birth**

***4a. Nulliparous women***				***4b.Multiparous women***			
	**Rate of intrapartum nulliparous referrals in midwifery practice (tertiles)**				**Rate of intrapartum nulliparous referrals in midwifery practice (tertiles)**		
	**T1 (low)**	**T2**	**T3 (high)**		**T1 (low)**	**T2**	**T3 (high)**
Instrumental birth rate	22.7%	26.5%	28.0	Instrumental birth rate	2.7%	2.8%	2.8%
Crude OR (95% CI)	1	1.23 (1.16 - 1.31)	1.33 (1.25 - 1.41)	Crude OR (95% CI)	1	1.06 (0.96 - 1.18)	1.05 (0.95 - 1.17)
Model 1 adjusted OR (95% CI)	1	1.22 (1.15 - 1.30)	1.33 (1.25 - 1.41)	Model 1 adjusted OR (95% CI)	1	1.07 (0.96 - 1.18)	1.04 (0.94 - 1.16)
Model 2 adjusted OR (95% CI)	1	1.22 (1.14 - 1.29)	1.31 (1.23 - 1.39)	Model 2 adjusted OR (95% CI)	1	1.05 ( 0.94 - 1.16)	1.01 (0.91 - 1.12)
Model 3 adjusted OR (95% CI)	1	1.21 (1.14 - 1.29)	1.31 (1.23 - 1.39)	Model 3 adjusted OR (95% CI)	1	1.05 ( 0.94 - 1.16)	1.01 (0.91 - 1.12)
Model 4 adjusted OR (95% CI)	1	1.08 (1.00 - 1.16)	1.05 (0.97 - 1.13)	Model 4 adjusted (OR) (95% CI)	1	0.99 (0.89 - 1.10)	0.87 (0.78 - 0.97)

model 1: adjustment for maternal age, gestational age, ethnic background urbanisation, SES.

model 2: model 1 + planned place of birth.

model 3: model 2 + size.

model 4: model 3 + any interventions (pain relief and/or augmentation).

Adjustment for differences in maternal characteristics did not change these results. Further adjustments by adding planned place of birth and practice size in the model did not change the associations either, although planned place of birth was a significant factor in the model. After adjustment for labour interventions the association was no longer statistically significant.

## Discussion

### Key findings

Our study showed a considerable variation in intrapartum referral rates between midwifery practices in the Netherlands. Women in practices with higher intrapartum referral rates received more often pharmacological pain relief and augmentation of labour. For nulliparous women the chance of an instrumental birth was also higher in these practices, even after adjustment for maternal and practice characteristics. The association between practice referral rate and instrumental birth was no longer significant after adjustment for pain medication and augmentation.

### Variation

Our results suggest that the wide range in intrapartum referral rates cannot easily be explained by maternal characteristics alone. Parity, the strongest predictor for referrals, does not explain the differences between the tertile groups, since we defined them based on the referrals of nulliparous women alone. The differences in other maternal characteristics were small and did not show a favourable case-mix of women in the practices in the lowest tertile. It is possible that unmeasured maternal characteristics are confounding the results, but it is unlikely that they can explain the wide range that we observed.

It is therefore probable that the midwifery practice or factors related to the practice are strong contributors to the variation in referral rates. The association between nulliparous and multiparous referral rates supports this suggestion: a practice that refers many nulliparous women also refers many multiparous women.

The variation was predominantly observed for non-urgent referrals during the first stage of labour. Differences between practices in the management of the first stage of labour can play a role, as well as midwives’ perception of the chance of a spontaneous vaginal birth [24]. Some other studies suggested as well that midwives’ risk perception or uncertainty are factors associated with referral or intervention decisions [25-28]. Furthermore, midwifery practices may vary in offering upright birth positions [29] or other non medical interventions that can help in reducing the need for obstetric interventions in physiological labour [30-32]. Variation in decision making is not unique for primary midwifery care. Variation is also observed in obstetric care, internationally and in the Netherlands. For instance, caesarean sections rates show considerable variation, even within a homogenous case-mix of nulliparous women with a term singleton vertex birth [33-36]. Obstetrician and hospital related factors contribute to this variation [33,37,38]. Since midwifery practices are working closely together with the local hospital and often share local multidisciplinary protocols with the obstetricians, this collaboration may also influence referral rates in their midwifery practice [18].

This kind of practice variation in health care has been a topic of concern since it was addressed by Wennberg and Gittelsohn in 1973 [39]. Much scientific effort was aimed at explaining such variation. Some variation can exist for good reasons such as differences in health needs or client preferences such as request for pain relief or planned place of birth. However, subjective factors such as a personal practice style has been suggested as an important source of variation, especially in areas where a solid scientific consensus is lacking [40]. Social and structural factors in the professional context play a role as well [41-44]. Reducing unwarranted variation is therefore complex. Glantz (2012) argues however that efforts to lower practice variation are worthwhile, since they may help in reducing unnecessary interventions in obstetrics in the US. Raising awareness by providing feedback to practitioners and hospitals about their own results is an essential element in this ambition [35]. This may also apply to lowering the variation in referral rates in primary midwifery care in the Netherlands.

### Referral: the first step in a cascade of interventions?

Our results show that a high referral rate is not without consequences for the women involved. Apart from the psychological consequences mentioned in the introduction, women also more often experienced a PPH as well as an instrumental birth in the practices with a higher referral rate. Both PPH and instrumental births are associated with an increased risk of serious maternal morbidity and mortality [45].

The higher occurrence of PPH is likely to be explained by the use of oxytocin for augmentation of labour, applied in the majority of births after referral [46]. The association between referral rate and instrumental birth is remarkable, even though an instrumental birth is always preceded by a referral. Our logistic regression analysis (model 4) suggests that receiving epidural pain relief and/or oxytocin for augmentation plays a mediating role in the association between a higher referral rate in the practice and a higher chance of an instrumental birth for nulliparous women. This finding is noteworthy. A Cochrane review showed an increased chance of an instrumental vaginal birth among women with epidural anaesthesia, but not of a caesarean section [47]. Augmentation with oxytocin had no significant effect on instrumental birth rates in another Cochrane review [48]. Moreover, authors who promote Active Management or its Dutch version Proactive Support of Labour [49,50] suggest that early intervention in case of a slow progress during the first stage is effective in preventing CS and instrumental birth. Our study does not support this assumption. Although we cannot give causal explanations, our results suggest that offering augmentation and/or pain relief increases the likelihood of instrumental birth, including Caesarean sections.

Since long, authors have warned for an accumulation - or cascade - of interventions; pain medication leads to a higher chance of augmentation or vice versa, which leads to an increased chance of an instrumental birth [51,52]. In primary midwifery care settings, a referral to obstetrician-led care can be seen as the first step in this cascade.

### Perinatal safety

The occurrence of a low Apgar score was rare, regardless of the referral rate, as can be expected in a low risk population. For an individual midwifery practice the incidence of an Apgar score < 4 of 0.1 - 0.3% means that this outcome occurs very infrequently, less than once in several years. The clinical significance of the somewhat higher occurrence of such a rare outcome in the lowest tertile group is difficult to interpret. However, this finding should be considered as a warning that a low referral rate should not be achieved at the cost of perinatal safety. It is noteworthy that in the lowest tertile group not only the percentage of non-urgent referrals is lower, but also the percentage of urgent referrals. This may indicate that urgent situations are not always recognized or not addressed adequately, although we can not examine this in the available database.

Perinatal safety should be safeguarded in all midwifery practices, not only in those with low referral rates. Perinatal audits are the best way to reflect in detail on individual cases of perinatal mortality and serious morbidity. Such audits were introduced nationwide successfully in the Netherlands in 2010 [53,54].

### Implications for practice

The wide variation in referral rates in the Netherlands is of concern. High intrapartum referral rates suggest that some of the referrals, especially non-urgent referrals during the first stage of labour, might have been unnecessary and therefore triggered avoidable interventions, including instrumental births and associated maternal morbidity. On the other hand, our results also confirm that achieving a low referral rate is no goal in itself. Perinatal safety should be warranted with timely referral to give access to obstetrical care.

An optimal range in referral rates care cannot be derived from our study. However, monitoring referral behaviour can help primary care midwives to maintain high quality midwifery care. Being aware of a high referral rate can stimulate midwives to reflect critically whether they can improve in supporting and promoting physiological childbirth, as described in the recent Lancet series [55]. At the other side of the spectrum, midwives with low referral rates may need to reflect on their ability to address emerging urgent situations in time. Independent midwifery practices should always incorporate the cooperation with the hospital in these reflections.

### Strengths and limitations of the study

A major strength of this study is that we had access to all records of low risk women in primary midwifery care during the study period. Using the combined database allowed us to use information from the midwifery registration as well as the obstetric registration. This improved the quality of our data on interventions in obstetrical care. However, the study has some limitations as well. It is based on routinely collected data. This type of study has an explorative character and does not allow for causal explanations. It is the first nationwide study relating referral rates in practices to birth outcomes in healthy low risk women. Controlling for maternal and practice characteristics in the performed analyses was however limited to variables available in the database. Interesting issues such as preferences of clients, organisational aspects of the midwifery practice or information about the collaboration with the hospitals referred to, could not be addressed.

## Conclusion

The wide variation between referral rates suggests that these differences between midwifery practices may not be fully explained by medical factors or client characteristics. A high intrapartum referral rate in a midwifery practice is associated with an increased chance of an instrumental birth, which appears to be mediated by the increased use of augmentation and medical pain medication. Midwives should be encouraged to critically evaluate their referral behaviour. A high referral rate in their practice may indicate that during the first stage of labour more interventions are applied than necessary. This may lead to a lower chance of a spontaneous vaginal birth and a higher risk on a PPH. However, a low referral rate should not be achieved at the cost of perinatal safety.
